# Dehydration and Cognition in Geriatrics: A Hydromolecular Hypothesis

**DOI:** 10.3389/fmolb.2016.00018

**Published:** 2016-05-12

**Authors:** Adonis Sfera, Michael Cummings, Carolina Osorio

**Affiliations:** ^1^Department of Psychiatry, Loma Linda UniversityLoma Linda, USA; ^2^Patton State HospitalPatton, USA

**Keywords:** dehydration, aquaporins, extracellular space, protein folding, protein conformational dynamics

## Abstract

Dehydration is one of the ten most frequent diagnoses responsible for the hospital admission of elderly in the United States. It is associated with increased mortality, morbidity and an estimated cost of 1.14 billion per year (Xiao et al., 2004; Schlanger et al., 2010; Pretorius et al., 2013; Frangeskou et al., 2015). Older individuals are predisposed to dehydration encephalopathy as a result of decreased total body water (TBW) and diminished sensation of thirst. We hypothesize that thirst blunting in older individuals is the result of a defective microRNA-6842-3p failing to silence the expression of the vesicular GABA transporters (VGAT) and alpha 7 cholinergic nicotinic receptors in the subfornical organ (SFO) of the hypothalamus. We hypothesize further that resultant dehydration facilitates protein misfolding and aggregation, predisposing to neurocognitive disorders. We completed a search of predicted microRNA targets, utilizing the public domain tool miRDB and found that microRNA-6842-3p modulates the SLC6A1 and CHRNA7 genes both of which were previously hypothesized to inhibit the thirst sensation by their action on SFO. The primary aim of this article is to answer two questions: Can prevention and correction of dehydration in elderly lower age-related cognitive deterioration? Can exosomal miR-6842 in the peripheral blood predict dehydration encephalopathy in elderly?

## Hydration and cognition

Dehydration is one of the most common medical problems in seniors diagnosed in 6.7% of hospitalized patients over the age of 65 (Warren et al., 1994). It leads to poor outcomes and increased health care expenditures. Novel studies reveal that if not prevented or treated promptly, dehydration results in longer intensive care unit (ICU) stay, higher hospital readmission rates and placement in long term facilities (Xiao et al., 2004; Frangeskou et al., 2015). On the other hand, preventing dehydration not only reduces healthcare expenditures, but also improves outcomes and the elderly patients' quality of life.

Dehydration is a contributing factor for delirium, a neurobehavioral syndrome recently demonstrated to be a strong risk factor for dementia (Inouye, 1998; Davis et al., 2012). It is therefore crucial to recognize and diagnose dehydration quickly, however at the present time there are no specific biological markers for this condition. Clinical signs, plasma osmolality and urine markers have poor specificity in elderly (George and Rockwood, 2004). For this reason potential epigenetic markers such as microRNA-6842-3p obtained from peripheral blood exosomes may contribute not only to early diagnosis, but also to prevention of dehydration.

Water is an essential body nutrient and its homeostasis is crucial for life. Early in the evolution, marine animals were surrounded by water, but survival on dry land required built-in, “portable” water (Warren et al., 1994). In humans, the muscle tissue is a genuine fluid reservoir, carrying over 80% of TBW (George and Rockwood, 2004).

The brain, in spite of being a highly lipophilic organ consists of 80% water (Tait et al., 2008). Most of the CNS intracellular water is stored in astrocytes. These cells are characterized by high aquaporin (AQP) expression which makes them four times more permeable to water than other brain cells, therefore true “brain cisterns” for times of water scarcity (Thrane et al., 2014). With the same token, because of their high AQP content, astrocytes are prone to pathological water retention and swelling. Novel studies demonstrate that astrocytes respond to peripheral dehydration by up-regulation of AQP-4 proteins on their end-feet processes probably in order to preserve water. For example, preclinical studies demonstrate that a hyperosmotic milieu induces AQP expression in astrocytes (Yang et al., 2013).

Overexpression of AQP-4 channels and augmented water intake transforms these cells into genuine “sponges” resulting in extracellular dehydration, extracellular space (ECS) hypovolemia. If severe enough this condition may turn into a medical emergency, dehydration encephalopathy or delirium.

The process of aging seems to undo the evolutionary advantage of “portable water” as elderly individuals are known to lose their fluid reservoirs by age-related decrease in both muscle mass and astrocyte density. For example, dehydration was demonstrated to accelerate the progression of AD which is also known to be associated with loss of astrocytes (Ogawa et al., 2011; Reyes-Haro et al., 2015; Rodríguez-Arellano et al., 2016).

It is well known that aging is associated with reduced acetylcholine (ACh) in the brain, but it is perhaps less emphasized that aging contributes to down-regulation of alpha7 nicotinic acetylcholine receptors (alpha7nAChR) (Utsugisawa et al., 1999; Akhmedov et al., 2013), rendering the CNS less responsive to ACh. This is significant for the sensation of thirst which is physiologically activated by ACh. Lower cholinergic activation predisposes to inflammation which is also involved in cognitive impairment. We discussed inflammation in the aging brain elsewhere and this subject will not be brought here (Sfera and Osorio, 2014). The alpha7nAChR are encoded by CHRNA7 gene which is subject to microRNA epigenetic regulation, including miR-6842.

Concerning the relationship between dehydration and impaired cognition nutrition studies demonstrate that a loss of only 1–2% of TBW may result in impaired cognitive performance; in elderly this percentage was shown to be even lower (Han and Wilber, 2013; Riebl and Davy, 2013). Furthermore, the link between hydration and cognition can be demonstrated by the neurocognitive disorders associated with up-regulation of AQP-4 expression primarily on asyrocytic end-feet (Table 1).

**Table 1:** **Disorders associated with cognitive deficit and AQP-4 up-regulation**.

**AQP-4/Cognitive deficit disorders**	**References**
Cerebral amyloid angiopathy	Foglio and Fabrizio, 2010; Moftakhar et al., 2010
Alzheimer's disease	Nagelhus and Ottersen, 2013; Lan et al., 2015
Parkinson's disease	Subburaman and Vanisree, 2011; Zhang et al., 2016
Multiple sclerosis	Tanaka et al., 2007
Neuromyelitis optica	Saji et al., 2013; Zhang et al., 2015
Traumatic brain injury	Hu et al., 2005
Cerebral ischemia	Zador et al., 2009
Epilepsy	Binder et al., 2012; Alvestad et al., 2013
HIV encephalitis	St. Hillaire et al., 2005
Progressive multifocal leukoencephalopathy	Aoki-Yoshino et al., 2005; Florence et al., 2012

Novel studies demonstrate that both dehydration and aging were associated with AQP-4 up-regulation, therefore it should not come as a surprise that aging and water loss go hand in hand (Trinh-Trang-Tan et al., 2003). Interestingly, several amyloid-binding, neuroprotective compounds were demonstrated to down-regulate AQP-4 expression, further demonstrating the role of water in amyloid pathology (Table 2).

**Table 2:** **Neuroprotective compounds associated with AQP-4 down-regulation**.

**AQP-4 down-regulation**	**References**
Rapamycin	Guo et al., 2014
Erythropoietin	Gunnarson et al., 2009; McCook et al., 2012
Curcumin	Laird et al., 2010; Wang et al., 2015
Purines	Morelli et al., 2010; Lee et al., 2013
Progesteron	He et al., 2014
Melatonin	Dehghan et al., 2013; Lin et al., 2013; Bhattacharyaa et al., 2014

In addition, neuroimaging studies in dehydrated elderly, show decrease in gray and white matter volume (Streitbürger et al., 2012). However, it is important to keep in mind that most brain volumetric studies rely on diffusion tensor imaging (DTI) which detects water anisotropy and is therefore highly dependent on the brain fluid dynamics (Meng et al., 2004; Nakamura et al., 2014).

## Water and protein misfolding disorders

Misfolded protein aggregates were shown to be involved in many human diseases, including neurocognitive disorders and diabetes type 2, but in spite of the increasing prevalence of these conditions, the reason proteins misfold is not completely understood.

Water has been known to play a major role in protein conformational dynamics (Lemieux, 1996; Phillips, 2002; Zhao et al., 2013). In order to become biologically active newly transcribed proteins must fold along specific axes like paper in the ancient Japanese art of origami (Collet, 2011; Chong and Ham, 2015). Recently it was demonstrated that water plays a crucial role in this process as it forms hydrogen bonds with the amino acid chains, facilitating their collapse into three dimensional molecular structures. In the presence of water, folding occurs almost instantly (140 ns), resulting in biologically active molecules available for chemical reactions at the opportune time (Sen and Voorheis, 2014; Vajda and Perczel, 2014). In the absence of hydration the folding process is significantly slower and the biomolecules may miss the timing of their reactions. This results in molecular overcrowding which predisposes to misfolding (Gregersen et al., 2006; Stoppini et al., 2009). Indeed, it was hypothesized by others that biomolecular crowding relative to the fluid volume is inductive of misfolding and aggregation (Tokuriki et al., 2004; Yerbury et al., 2005).

Novel studies in protein conformational dynamics demonstrate that both protein misfolding, their repair and removal can take place in the intra and the extracellular compartment. The chance of protein misfolding is higher in the extracellular space (ECS) which is a rougher environment exposing these biomolecules to a higher degree of shear and tear (Ker and Chen, 1998; Genereux and Wiseman, 2015). For this reason, we focus our study on the ECS where hypovolemia may facilitate protein misfolding and aggregation.

**Figure 1:**
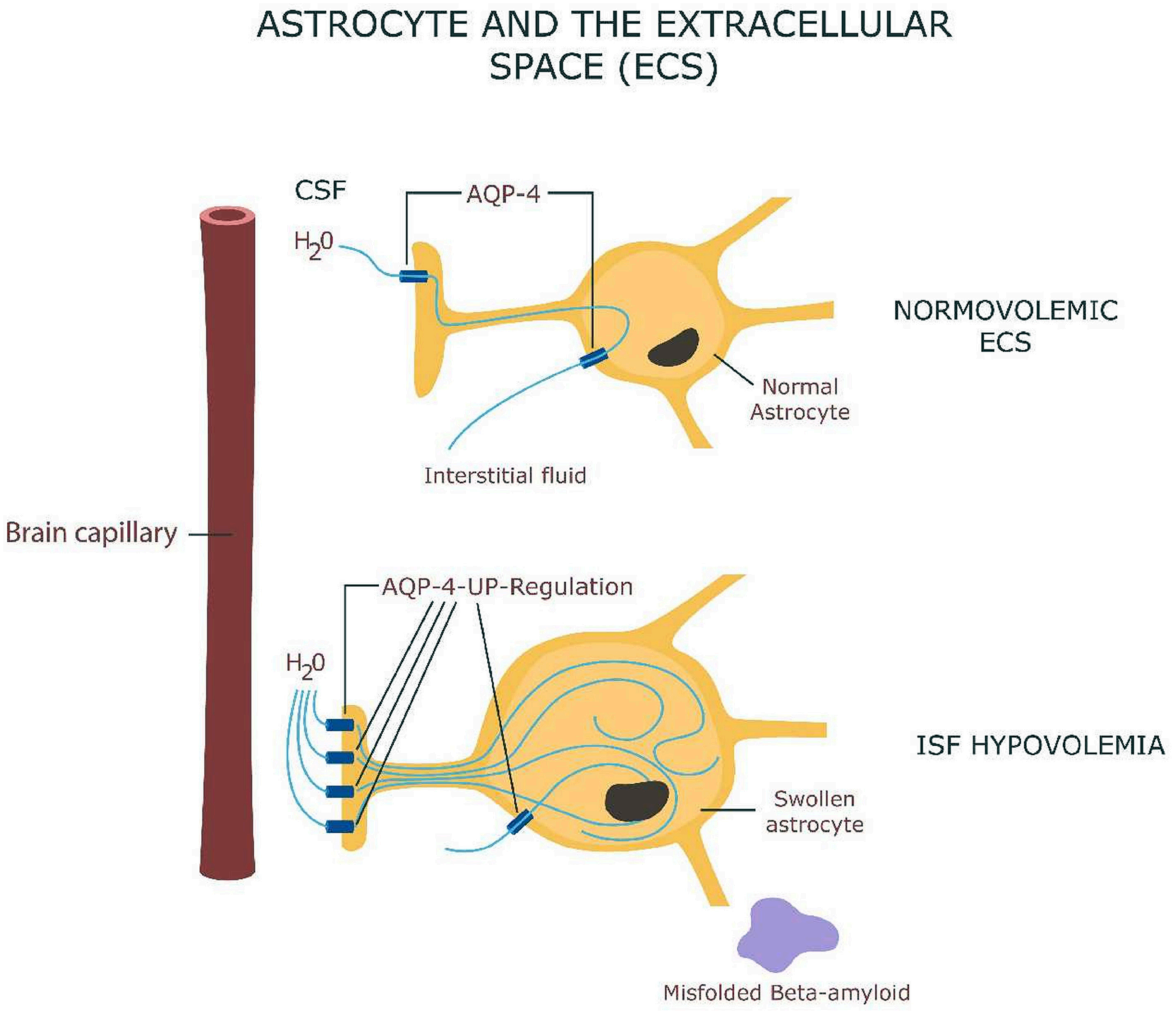
**Astrocyte swelling as a result of AQP-4 channels up-regulation with interstitial fluid hypovolemia and beta-amyloid misfolding**.

It was hypothesized that adequate water circulation via aquaporin (AQP) channels is essential for clearing beta amyloid and for preventing its build-up characteristic for Alzheimer's disease (AD) (Figure 1). The glymphatic system paradigm suggests that insufficient amyloid clearance and its subsequent aggregation is the result of impaired water movement (Xie et al., 2013). This model, however pays less attention as to why proteins misfold in the first place.

The hydromolecular hypothesis is therefore complementary to the glymphatic model, but also differs from it by elevating water from an inert medium to an active participant in cognition (via protein folding) (Levy and Onuchic, 2006). This hypothesis raises another interesting question: do proteins participate in information processing directly?

Novel studies in neuroscience demonstrate that proteins participate in cognition by their ability to access logic gates, the elementary building blocks of digital circuits (Qi et al., 2013). These molecules are endowed with abilities to adaptively change their shapes in Transformers-like fashion, assembling and disassembling in response to electronic signals or electromagnetic fields (Kidd et al., 2009; Ausländer et al., 2012). For example, proteins were shown to assemble in the neuronal post-synaptic membrane into heteroreceptor complexes which may engender memory “bar codes” (Fuxe et al., 2007; Chen et al., 2012). Calcium-calmodulin-dependent kinase III, a component of neuronal microtubules, was hypothesized to store long term memory by reorganizing its spatial structure in response to synaptic activity (Smythies, 2015). Interestingly, water plays a major role in this model. Several studies revealed that dendritic spine biomolecules may play a crucial role in associative memory as they endow the neural circuits with Boolean logic (Craddock et al., 2012; De Ronde et al., 2012; Qi et al., 2013). Furthermore, proteins are endowed with Lego-like abilities to interlink, engendering large intra and extracellular biomolecular networks with hypothesized roles in cognition (Chen et al., 2012; Mancuso et al., 2014). In light of this data we believe that alteration of the normal protein conformation may impair cognition directly, rather than indirectly by damaging synapses and neurons which is the traditional view.

## Epigenomic regulation of the subfornical organ (SFO)

Elderly individuals are prone to dehydration as a result of blunted thirst sensation and loss of TBW as discussed above (Cowen et al., 2013; Hooper et al., 2014). Recent preclinical data reveal that the subfornical organ (SFO) of the hypothalamus functions as a “thirst center” in the mammalian brain, regulating the basic instinct of water intake (Oka et al., 2015). Since the SFO lacks a blood-brain-barrier (BBB) it may be well positioned to detect peripheral dehydration and respond to it by increasing the sensation of thirst lowering water output. The SFO contains sensitive osmoreceptors which convert peripheral changes in osmolality into an excitatory neuronal signal, triggering both the sensation of thirst and the release of arginine vasopressin (AVP) by the posterior pituitary (Azizi et al., 2008).

It was recently demonstrated that the SFO contains both excitatory and inhibitory neurons which can be activated by the ECS water volume and osmolality (Oka et al., 2015). ECS hypovolemia activates the SFO excitatory neurons (which express ETV-1 transcription factor), triggering thirst. ECS normovolemia, on the other hand activates the SFO inhibitory neurons (which express the vesicular GABA transporter (VGAT)], inhibiting the sensation of thirst.

These genetically distinct neuronal groups may explain both dehydration and psychotic polydipsia. For example, excessive activation of excitatory, or failure to activate inhibitory SFO neurons may result in psychotic polydipsia. The opposite may be true in dehydration.

**Figure 2:**
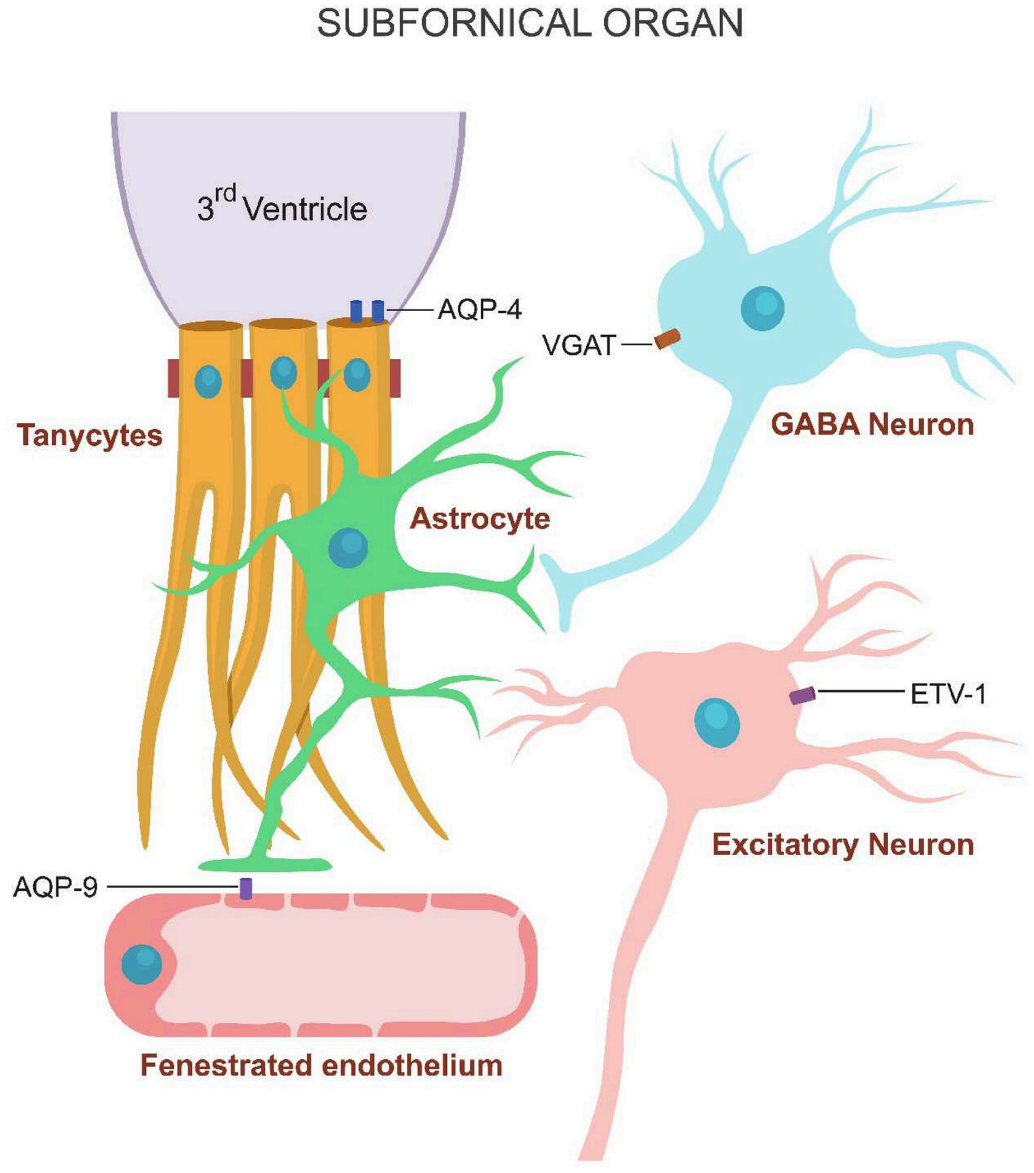
**Water channels expressed on the SFO cells**. Inhibitory GABAergic neurons express VGAT, astrocytes AQP-9 and tanycytes AQP-4.

Several prior studies revealed that the sensation of thirst may also be activated by the stimulation of SFO neuronal cholinergic receptors. The SFO neurons express both nicotinic and muscarinic cholinergic receptors, while the SFO astrocytes express only alpha 7- nAChRs (Honda et al., 2003; Tanaka, 2003; Ono et al., 2008). Age-related paucity of these receptors interferes with ACh activation of the thirst sensation. The glial water channels consist of AQP-9 expressed by astrocytes and AQP-4 expressed by tanycytes (Figure 2).

In addition to decreasing the expression of alpha 7 nAChRs, the aging process was documented to augment the expression of AQP channels on astrocytic end-feet as part of an age-related senescence-associated secretory phenotype (SASP). SASP is characterized by low grade inflammation, increased accumulation of misfolded protein aggregates and astrocyte swelling induced by AQP up-regulation (Picciotto and Zoli, 2002; Salminen et al., 2011; Akhmedov et al., 2013).

Peripheral dehydration was demonstrated to alter the expression of several SFO- related genes (Hindmarch et al., 2008). One of these genes is SLC6A1 which expresses VGAT on the cellular membranes of the SFO inhibitory neurons.

**Figure 3:**
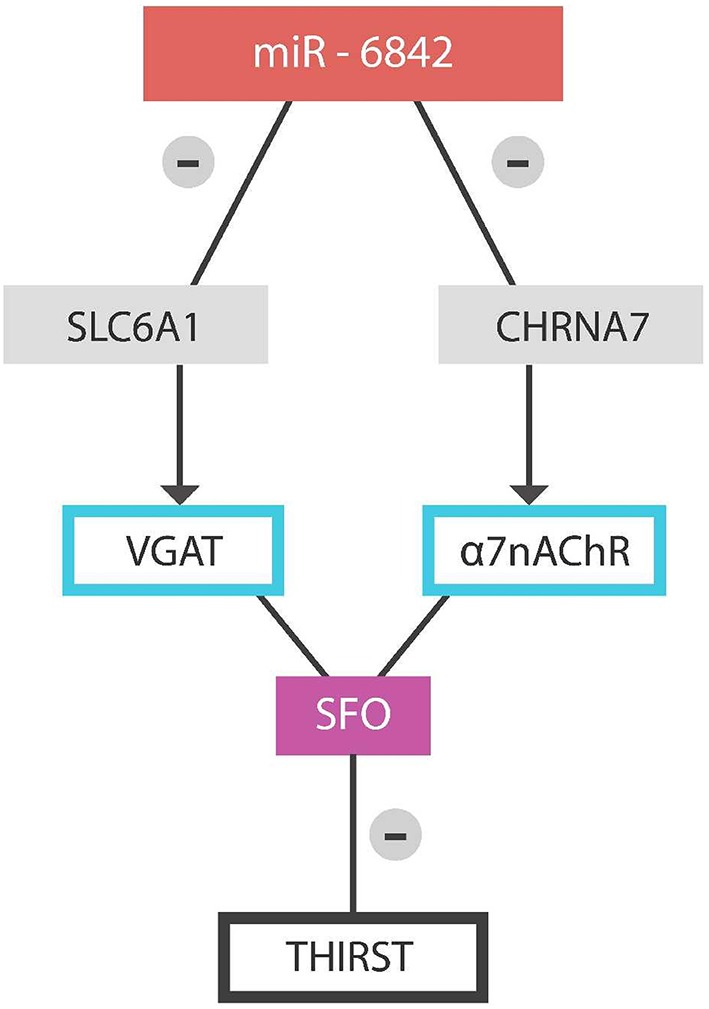
**Physiologically, microRNA-6842 silences SLC6A1 and CHRNA7 genes, activating the sensation of thirst**.

Method: we conducted a search of miRDB, a public online database for microRNA target prediction and functional annotations. The targets in miRDB are predicted by the bioinformatics tool, MirTarget. MirTarget was developed by analyzing thousands of miRNA-target interactions from high-throughput sequencing experiments. We searched the human database for the genes of interest SLC 32A1 and CHRNA 7, coding for VGAT and alpha 7 nicotinic cholinergic receptors respectively. We conducted a separate search for each of the two genes by utilizing the gene symbol SLC 32A1 and CHRNA 7. The results revealed that 131 microRNAs modulate the SLC 32A1 gene and 57 microRNAs the CHRNA 7 gene. Analyzing this data, miR by miR we found one common microRNA modulating both genes, the miR-6842 (Figure 3).

A dysfunctional miR-6842 may fail to silence the SLC6A1 gene, preventing inhibition of the SFO GABAergic neurons with resultant thirst blocking. The same is achieved via failure to inhibit the CHRNA-7 gene, thus preventing ACh-induced thirst.

## Conclusions

The hydromolecular hypothesis endeavors to explain the relationship between dehydration and decreased cognition in elderly as resulting from protein misfolding and aggregation in the context of low interstitial fluid volume (ECS hypovolemia). Defective proteins may affect cognition either directly via impaired information processing in the brain biomolecular networks, or indirectly via neuronal and synaptic damage, or both.

MicroRNA-6842 may constitute a biological marker with predictive value for dehydration encephalopathy in elderly as it regulates two genes involved in the sensation of thirst.

## Author contributions

All authors listed, have made substantial, direct and intellectual contribution to the work, and approved it for publication.

### Conflict of interest statement

The authors declare that the research was conducted in the absence of any commercial or financial relationships that could be construed as a potential conflict of interest.
